# Management of refractory cervical anastomotic fistula after esophagectomy using the pectoralis major myocutaneous flap

**DOI:** 10.1016/j.bjorl.2020.05.009

**Published:** 2020-06-15

**Authors:** Lifei Deng, Yan Li, Weixiong Li, Muyuan Liu, Shaowei Xu, Hanwei Peng

**Affiliations:** a Cancer Hospital of Jiangxi Province, Department of Head and Neck Surgery, Nanchang, Jiangxi, China; b Cancer Hospital of Shantou University Medical College, Department of Gynecology, Shantou, Guangdong, China; c Chaozhou People's Hospital, Department of Head and Neck Surgery, Chaozhou, Guangdong, China; d Cancer Hospital of Shantou University Medical College, Department of Head and Neck Surgery, Shantou, Guangdong, China

**Keywords:** Esophagectomy, Anastomotic leakage, Reconstruction, Pectoralis muscle, Myocutaneous flap

## Abstract

**Introduction:**

A refractory cervical anastomotic fistula which postoperatively remains unhealed for more than 2 months under conservative care severely impacts the quality of life of the patient and potentially leads to anastomotic stricture after the fistula heals. It is widely accepted that, to avoid this complication, refractory cervical anastomotic fistulas should undergo more aggressive treatments. However, when and which surgical intervention should be considered is unclear.

**Objective:**

This study was designed to evaluate the role of the pectoralis major myocutaneous flap in the management of refractory cervical anastomotic fistulas based on our experience of 6 cases and a literature review.

**Methods:**

Six patients diagnosed with refractory cervical anastomotic fistula after esophagectomy treated using pectoralis major myocutaneous flap transfer were included in the study. The clinical data, surgical details, and treatment outcome were retrospectively analyzed.

**Results:**

All patients survived the operations. One patient who had a circumferential anastomotic defect resulting from surgical exploration developed a mild fistula in the neo-anastomotic site in the 5th postoperative day, which healed after 7 days of conservative care. This patient developed an anastomotic stricture which was partially alleviated by an endoscopic anastomotic dilatation. All the other 5 patients had uneventful recoveries after operations and restored oral intake on the 10th–15th days after operation, and they tolerated normal diets without subsequent sequelae on follow-up. One patient developed both local and lung recurrence and died in 15 months after operation, while the other 5 patients survived with good tumor control during the follow-up of 25–53 months.

**Conclusion:**

The satisfactory treatment outcome in our study demonstrates that pectoralis major myocutaneous flap reconstruction is a reliable management modality for refractory cervical anastomotic fistulas after esophagectomy, particularly for those patients who experienced persistent fistulas after conservative wound care and repeated wound closures.

## Introduction

Anastomotic fistula (AF) is one of the most common and troublesome postoperative complications after esophageal reconstruction in surgical treatment of esophageal cancer. Although modern surgical techniques, devices and perioperative management protocols have improved the safety of esophagectomy,^1^, ^2^, ^3^, ^4^ the incidence of this postoperative morbidity has stayed stubbornly high, up to 25%.^5^ AF not only results in a prolonged hospital stay, but also occasionally causes life-threatening infections, e.g. pyothorax and mediastinitis.^6^, ^7^ It has been reported that AF after esophagectomy is mainly associated with the following three surgical factors: the lack of serosa, the presence of a segmental blood supply and the presence of tension.^8^, ^9^ Many other factors such as neoadjuvant chemoradiotherapy, anatomic site of the anastomosis, diabetes mellitus, body mass index, age, congestive heart failure, hypertension, renal insufficiency, smoking, and even superior thoracic aperture size have been reported to contribute to the risk of AF.^10^, ^11^, ^12^

The incidence of the cervical AFs is much higher than that of the thoracic AFs.^13^ Fortunately, most of the cervical AFs are curable after conservative treatments such as wound debridement, irrigation, and drainage.^14^ However, a small number of cervical AFs become refractory (refractory cervical anastomotic fistula – RCAF), which is defined as noncurative anastomotic site-cutaneous fistula that has existed longer than 2 months under conservative medical care.^9^ Even without life-threatening complications, a persistent unhealed cervical AF not only severely impacts the quality of life of the patient, but also potentially leads to anastomotic stricture after the fistula heals.^15^, ^16^ It is widely accepted that patients with RCAFs should undergo more aggressive treatments, involving covered stents, primary closure and vascularized pedicle tissue flaps. Nevertheless, the covered stent insertion into the cervical esophagus often causes strong pharyngeal discomfort or stent migration, which ultimately results in treatment failure.^17^ Although custom-designed stents have been successfully employed in the treatment of cervical anastomotic leakage after esophagectomy,^18^ their effectiveness requires more studies for confirmation, and such custom-designed stents are not commercially available. Simple primary closure is often associated with leakage relapses due to poor local tissue vitality and infection. To our knowledge, only a few studies have demonstrated that vascularized pedicled tissue flaps, such as sternocleidomastoid flaps and pectoralis major myocutaneous flaps (PMF) could be used safely and effectively to cure anastomotic complications.^19^, ^20^, ^21^, ^22^, ^23^, ^24^ However, the role of PMF in management of patients with RCAF is still unknown due to limited case series. In this retrospective study, the efficacy and surgical details in the management of 6 patients with RCAF after esophagectomy for their esophageal cancer using PMF is discussed, and a literature review was performed.

## Methods

### Data collection and study approval

Clinical data were collected based on our institutional database (Cancer Hospital of Shantou University Medical College). Demographic details, indications, pathological stages (AJCC TNM staging system, 2012), type of esophagectomy, comorbidity, previous treatment, and treatment outcomes were recorded.

This study was approved by the Medical Ethics Committees of Cancer Hospital of Shantou University Medical College (approval no. 2020001), and was performed in accordance with the ethical standards of the Helsinki Declaration of 1975 and all subsequent revisions. All persons mentioned in the paper gave written informed consents for their data/images to be used for study and publication.

### Definition of RCAF

Definition of RCAF referred to uncurable anastomotic site-cutaneous fistulae that had been present for more than 2 months in patients under conservative medical care or after primary wound closure(s).

### Demographic data

From June 2015 through December 2017, a total of 6 patients diagnosed with RCAF following esophagectomy were referred to the department of the corresponding author for care. Demographic data of these patients was shown in Table 1. Their ages ranged from 47 to 68. Four were male and 2 were female. None of these 6 patients underwent preoperative chemotherapy or chemoradiotherapy. In 1 patient (pt. no. 2) the gastric tube was passed to the neck via a retrosternal route, and the other 5 patients had the tubes directed to the neck via a posterior mediastinal route. The cervical AFs were confirmed in the 2nd to 5th postoperative day, and all the fistulas remained unhealed for more than 2 months. Endoscopic stent insertion had been attempted once and failed in patient no. 4. Simple wound closures had been attempted 4 times and failed in patient no. 3. The curative surgeries involving a PMF reconstruction procedure were performed in 2–8 months after the initial esophagectomy. Informed consents of patients in this study were obtained before operations.

**Table 1:** Demographic data and clinic pathologic breakdowns of the 6 patients enrolled in this study.

Case no.	Age	Sex	Subsite	Stage	Comorbidities	Main techniques used in esophagectomy	The time fistula occurred	Repair time (months)	Followup time (month)	Result
1	47	Male	MTE	T3N1M0	None	Left thoracotomy, two-field lymphadenectomy, hand-sewn anastomosis, posterior mediastinal route	Severe anastomotic site bleeding 8 h postoperatively, fistula in 2nd postoperative day	3	27	Postoperative radiation performed, survive, normal oral intake
2	68	Male	MTE	T2N0M0	HT, HL	Right thoracotomy, three-field lymphadenectomy, mechanic anastomosis, retrosternal route	2nd POD	3	25	Survive, oral intake, tolerate soft diet
3	56	Male	MTE	T2N0M0	None	Right thoracoscopy, three-field lymphadenectomy, mechanic anastomosis, posterior mediastinal route, endoscopic	5th POD	8	53	Survive, normal oral intake
4	51	Female	UTE	T1N0M0	HL	Left thoracotomy, hand-sewn anastomosis, posterior mediastinal route	3rd POD	2	42	Survive with normal oral intake
5	60	Male	MTE	T2N0M0	HT, DM	Right thoracoscopy, two-field lymphadenectomy, mechanic anastomosis, posterior mediastinal route	2nd POD	3	15	Normal oral intake, died in 15 months after operation due to tumor recurrence
6	49	Female	MTE	T2N0M0	None	Left thoracotomy, two-filed lymphadenectomy, hand-sewn anastomosis, posterior mediastinal route	3rd POD	3	34	Survive, normal oral intake

MTE, middle thoracic esophagus; UTE, upper thoracic esophagus; HT, hypertension; HL, hyperlipemia; DM, diabetes mellitus; PO, postoperative day.

### Typical cases and surgical procedure

Patient no. 1 was male, 47 years old, who underwent esophagectomy and gastric pull-up with cervical anastomosis via left thoracotomy for his T3N0M0 mid-thoracic esophageal squamous cell carcinoma in a tertiary hospital. The patient experienced severe bleeding in the anastomotic site 8 h after surgery and underwent surgical exploration for hemostasis, with blood transfusion of 2000 mL. However, the patient presented with bloody drainage of 500–700 mL per day for 4 days, and on the 5th postoperative day, purulent and salivary drainage mixed with blood was seen. Fortunately, no pyothorax and mediastinitis occurred. Nutrition support with both intravenous infusion and nasogastric feeding, antibiotics, and wound drainage were adopted and the patient was discharged on the 30th postoperative day with an unhealed cervical fistula which had persisted for 2 months despite careful wound care and two attempts at simple surgical wound closure. The patient was then referred to our department (Department of Head and Neck Surgery, Cancer Hospital of Shantou University Medical College) for further management.

**Figure 1:**
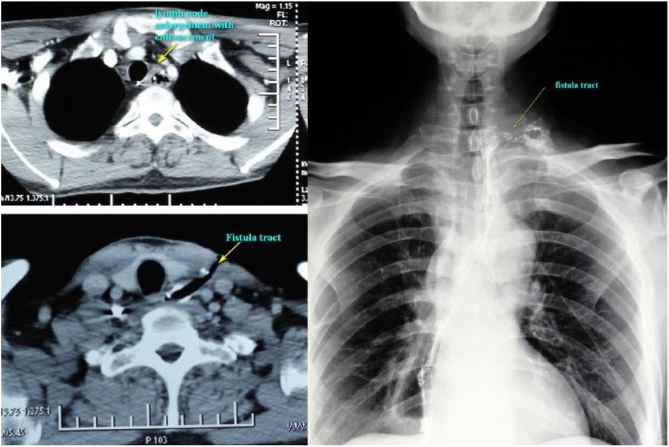
Enhanced MRI/CT scan and barium swallow exam demonstrated a fistulous tract from the anastomotic site to the lateral neck with enlarged lymph nodes found in level IV.

Enhanced MRI/CT scan and barium swallow exam demonstrated a fistulous tract from the anastomotic site to the lateral neck with enlarged lymph nodes found in level IV (Fig. 1). After informed consent was obtained, surgical exploration and fistula repair with PMF were performed.

**Figure 2:**
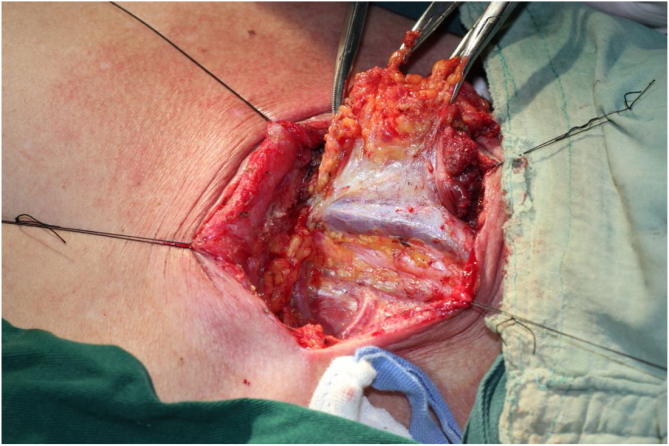
After elevation of the platysma flap, the sternocleidomastoid muscle was exposed and its lower half was removed to make space for the muscular pedicle of PMF.

**Figure 3:**
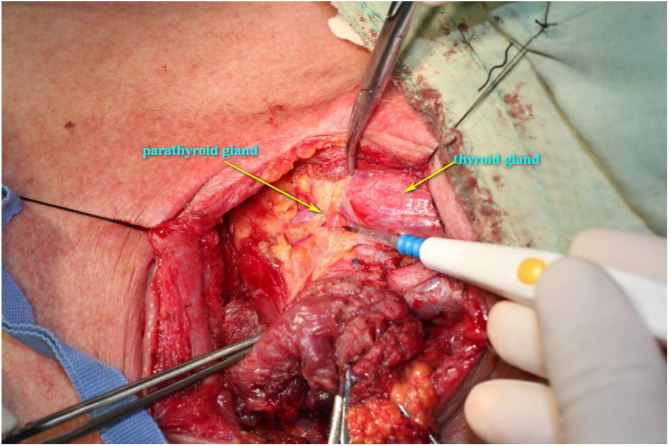
The left strap muscles were removed and the thyroid gland was exposed with identification of the upper and the lower parathyroid glands, as well as recurrent laryngeal nerve.

**Figure 4:**
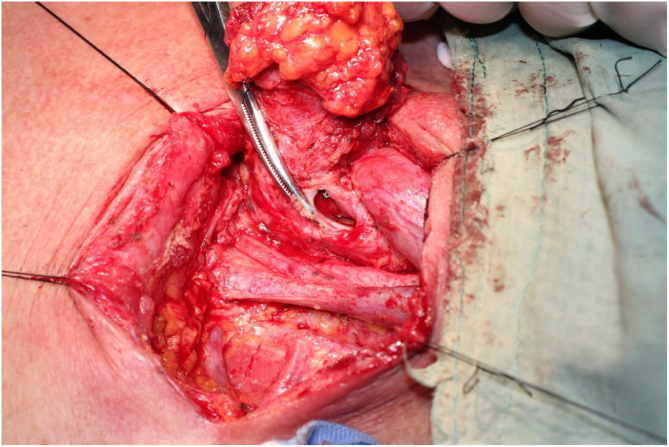
The gastro-esophageal anastomotic site was reached and opened.

**Figure 5:**
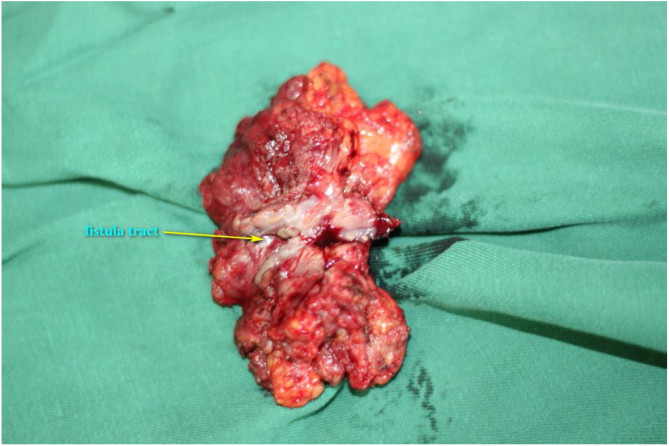
The granulation tissue embedding the fistula tract with part of the gastroesophageal stump was removed enbloc.

The procedure was performed under general anesthesia with the patient in supine position with the neck extended. An incision was made along the surgical scar with extension to the lateral neck. After elevation of the platysma flap, the sternocleidomastoid muscle was exposed and its lower half was removed to make space for the muscular pedicle of PMF, and level III–V dissection was performed (Fig. 2). The left strap muscles were removed and the thyroid gland was exposed with identification of the upper and the lower parathyroid glands, as well as the recurrent laryngeal nerve (Fig. 3). After retracting the thyroid gland medially, the gastro-esophageal anastomotic site was reached and opened (Fig. 4). The granulation tissue embedding the fistula tract with part of the gastroesophageal stump was removed enbloc (Fig. 5) to make a fresh wound ready to be repaired.

**Figure 6:**
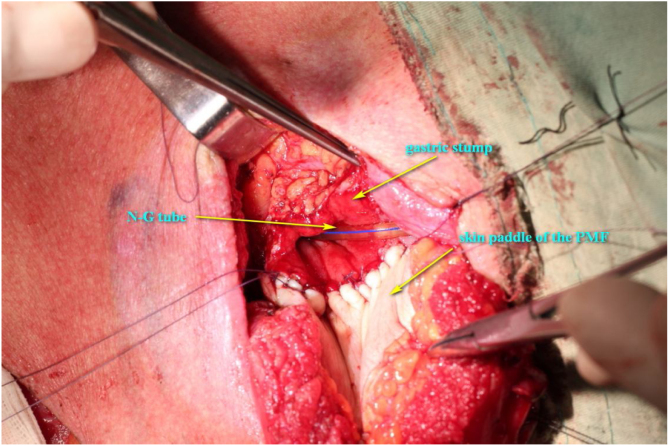
The defect of the anastomotic site was repaired using the skin paddle with its skin side toward the lumen.

**Figure 7:**
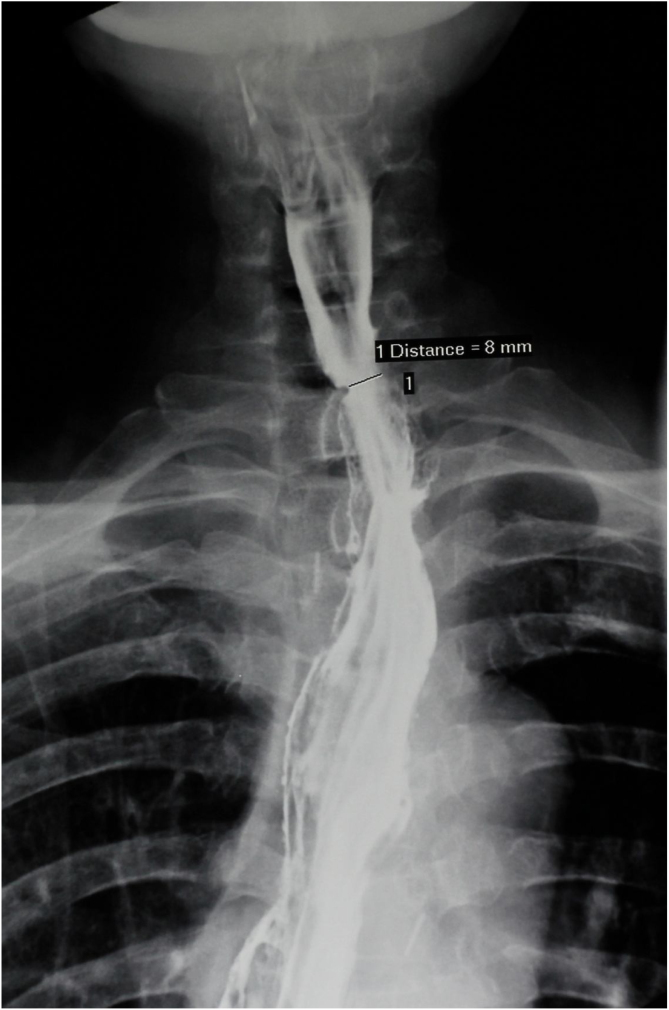
Barium swallowing X-ray 10 months later demonstrated a smooth alimentary tract without any anastomotic stricture.

The PMF was harvested using our established technique.^25^ In brief, it was harvested as a pedicle-skeletonized flap, with its skin paddle (7 cm × 5 cm) caudally and medially to the areola, including the third intercostal perforator, preserving the upper one third of the pectoralis major muscle. The clavipectoral fascia was divided and the harvested flap was passed via a submuscular tunnel over the clavicle. The defect of the anastomotic site was repaired using the skin paddle with its skin side toward the lumen (Fig. 6). A draining tube was placed and both wounds were closed directly without difficulty. The postoperative recovery was uneventful and the patient returned to liquid oral intake in 10 days after the operation. Postoperative adjuvant radiotherapy was administered in 4 weeks after the operation due to the pathological finding of 2 lymph node metastases. The barium swallow X-ray exam 10 months later demonstrated a smooth alimentary tract without anastomotic stricture (Fig. 7). The patient tolerated a normal diet during follow-up.

Patient no. 3–6 had a similar medical history and underwent a similar surgical procedure and had an uneventful recovery as patient no. 1.

Patient no. 2 underwent a right thoracotomy esophagectomy with gastric conduit sent to the neck via a retrosternal route for his mid-thoracic esophageal squamous cell carcinoma staged T2N0M0 in a tertiary hospital. A cervical anastomotic fistula occurred on the 2nd postoperative day with purulent and salivary drainage. No pyothorax and mediastinitis developed and the patient was discharged on the 15th postoperative day with an unhealed neck fistula, receiving nutrition via nasogastric tube feeding. An MRI scan 3 months after the operation demonstrated a cervical anastomotic fistula and stricture. With the informed consent of the patient and family, we performed the surgical exploration and planned a PMF reconstruction of the anastomotic site.

Surgical procedure performed was similar to patient no. 1. Severe adhesion and granulation tissue surrounding the fistula tract was found in exploration (Fig. S1). After breaking down the anastomotic site, necrosis of the anterior tubed gastric wall was readily seen extending to the retrosternal space, and the anastomosis site was completely closed by stricture and accommodated only the nasogastric tube (Fig. S2). Subsequent sternotomy facilitated identification of the healthy stump of the gastric wall. After removal of the anastomotic fistula and the necrotic gastric wall, a circumferential defect of alimentary tract 8 cm in length remained. A PMF with skin paddle larger than previously designed (12 cm × 8 cm) was harvested and tubed to reconstruct the defect (Fig. S3). Purulent drainage was noticed 5 days after operation, and this subsided after wound drainage and intravenous infusion of antibiotics. Oral liquid intake was successfully attempted on the 22nd postoperative day. However, he developed an anastomotic stricture and underwent an anastomotic dilatation which subsequently led to tolerance of soft diet. The patient refused further intervention afterward. Six months after operation, barium contrast X-ray demonstrated a mild anastomotic stricture with a diameter of 0.4 cm in the anastomotic site. At the last follow-up (Fig. S4), he was tolerating a soft diet.

## Results

### Surgical outcomes

All patients survived the operations. Patient no. 2 developed a mild fistula in the neo-anastomotic site, and the patient developed an anastomotic stricture which was partially alleviated by an endoscopic anastomotic dilatation. This patient tolerated a soft diet on follow-up. All the other 5 patients had uneventful recoveries after operations and restored oral intakes on the 10th–15th days after operation. All tolerated normal diets without subsequent sequelae on follow-up. Patient no. 1 was upstaged due to pathologic identification of lymph node metastases in level IV, and adjuvant radiotherapy was administered 4 weeks after operation. Patient no. 5 developed both local and lung recurrence and died 15 months after operation, while the other 5 patients survived with good tumor control during the follow-up of 25–53 months.

## Discussion

A majority of the cervical AFs resulting from esophagectomy are not fatal and they are curable after conservative wound care, including wound debridement, irrigation, and drainage. However, a small number of these patients with AFs are refractory under the conservative care regimen and they need more aggressive treatments, such as covered stent and primary closure. Covered stent has been reported to be effective in the management of patients with thoracic anastomotic fistula and esophagotracheal fistula.^26^, ^27^, ^28^ However, traditional esophageal stents are indicated for the thoracic AFs rather than the cervical AFs because of the potential patient intolerance or stent migration as the residual esophagus is too short for the placement of the stents, and the stent migration inevitably leads to the failure to stop the leakage.^18^ Custom-designed stents have been developed and reported to be effective for cervical anastomotic fistula, however, the study sample size is quite small, and this novel stent is unavailable commercially.^18^ In our study series, covered stent was attempted and failed in only 1 patient (pt. no. 4) due to stent migration and patient intolerance.

Primary surgical closure is often attempted after failure of conservative wound care and/or stent placement. However, simple primary closure of the wound only works for a certain number of patients, and the remaining patients often develop recurrence because of the necrosis, unhealthy granulation tissue, fibrosis, and wound infections. Repeated fistulation and wound closure have a negative impact on the quality of life of the patient. Furthermore, healing of long-term anastomotic fistulas could subsequently result in anastomotic strictures in some cases.^15^, ^16^, ^29^ Patient no. 3 in this series had 4 primary closures during his postoperative period of 8 months, and he had to rely on a nasogastric tube feeding during this period. In this situation, radical surgery intervention should be considered in order to shorten the rehabilitation period, to improve the quality of life, and to prevent the subsequent anastomotic strictures.

The purpose of surgical intervention is to debride the unhealthy tissue of the fistula orifice and fistula tract to create a fresh wound which will be repaired by a well vascularized flap to facilitate wound healing and prevent the possible subsequent anastomotic stricture. In fact, the main etiology of anastomotic fistulation is associated with blood supply compromise of the gastric stump and/or the cervical esophageal stump leading to poor healing of the anastomosis and subsequent leakage.^8^, ^9^, ^13^, ^30^ To deal with the problem, well-vascularized tissue should be used to facilitate would healing. Theoretically, both pedicled flaps and free flaps are management options. However, free flaps are rarely selected due to the poor general status of the patient and the high risk of failure in the non-optimal recipient sites, whereas pedicled flaps, including PMF and sternocleidomastoid muscle flaps, are reliable donor sites in that they are anatomically close to the fistula and the flaps are easy to harvest with their feeding vessels intact. However, a literature review generated only a few reports on the use of pedicled flaps for refractory cervical fistula after esophagectomy.^19^, ^20^, ^21^, ^22^, ^23^, ^24^ The patient involved, types of flaps used, and treatment outcome are summarized in Table 2. Although almost all patients involved in the studies had a satisfactory treatment outcome with regard to anastomotic fistula or stricture, there are only 20 cases reported across these 6 studies. We believe that this is possibly due to the lack of communications between thoracic surgeons and head and neck surgeons.

**Table 2:** Summary of reports on management of patients with refractory fistula using vascularized pedicle flaps.

Year	Author	Type of flap	Summary of the involved cases	Outcome
1998	Heitmiller, RF^24^	PMF	Cervical anastomotic stricture (*n* = 2), anastomotic leakage (*n* = 1)	Small leakage (*n* = 2), seroma in the donor site (*n* = 1); all healed with good function outcome
1998	Williums, JK^23^	PMF	Retrosternal esophago-colol anastomotic leakage (*n* = 1)	Healed with good function outcome
2006	Hirao, M^22^	PMF with T-tube drainage	Cervical esophageal anastomosis after esophagectomy for cancer using a jejunum interposition (*n* = 1)	Healed with good function outcome
2010	Morita, M^21^	PMF	Anastomotic leakage after esophageal reconstruction via the subcutaneous route (*n* = 6)	Primarily healed (*n* = 5), leakage recurrence and healed after conservative wound care (*n* = 1)
2014	Nakajima, M^20^	Sternocleidomastoid muscle flap	Cervical anastomotic leakage after esophagectomy for esophageal cancer (*n* = 8)	Healed with good function result
2015	Yin, K^19^	PMF	Colon conduit necrosis and cervical-oesophageal discontinuity (*n* = 1)	Healed with good function result

Since its introduction by Ariyan in 1979, the PMF has been used as a workhorse flap for the reconstruction of the head and neck defects because of its ease of harvest, abundant soft tissue volume, large skin paddle, generous blood supply, relative versatility, considerable reliability, and short operating time.^31^, ^32^ Even in the era of free flap reconstructions, its role in reconstructive surgery is still irreplaceable.^25^, ^31^, ^33^, ^34^ In addition to primary head and neck reconstruction and thoracic wall defect reconstruction, the PMF also plays a valuable role in salvage surgeries, e.g. salvage reconstruction after free flap failure and management of the hypopharyngeal fistulas.^25^, ^35^

We presumed that this flap is an appropriate donor site for the repair of RCAF based on the following hypotheses: (1) The abundant muscle paddle with reliable blood supply could facilitate dead space debridement, wound healing, and protection of the carotid vessels; (2) The skin paddle could be used as a patch to repair the anastomotic conduits, which would avoid subsequent anastomotic strictures; (3) The skin paddle could be tubed to reconstruct the circumferential or near circumferential defect after complete removal of the fistula tract and its surrounding tissues as well as the unhealthy gastroesophageal stump; (4) Easy flap harvesting would make it suitable for debilitated patients with poor health status.

We believe that the sternocleidomastoid muscle flap may also be a good option for the management of patients with cervical RCAF, particularly for those with intact lower necks and small fistula tracts. However, we chose PMF as the donor site rather than sternocleidomastoid muscle flap because: (1) We have developed a advanced harvesting technique for PMF and have never experienced total flap necrosis in our practice^25^; (2) The pedicle skeletonization and preservation of the upper two-thirds of the pectoralis major facilitates tailoring of the muscle paddle and the skin paddle to minimize the bulge in the neck after the lower half of the sternocleidomastoid muscle and level IV–VI soft tissues are removed; (3) The aforementioned natural features of this flap. In our study, Five out of 6 patients had an uneventful recovery and restored oral intake with anormal diet. Only patient no. 2 who had a circumferential defect that required a tubed PMF developed a mild fistula after reconstruction. This patient had a subsequent anastomotic stricture which was partially alleviated by endoscopic dilatation.

## Conclusion

The satisfactory treatment outcome in our study demonstrates that PMF reconstruction is a reliable management modality for RCAFs after esophagectomy, particularly for those who failed after conservative wound care and repeated wound closures. However, this conclusion is limited to a small number of patients under the study. More studies are needed to support our conclusion.

## Funding

This work was supported by the Guangdong Provincial Grant for Scientific and Technologic Project, China (Grant no. 2019-113-67).

## Conflicts of interest

The authors declare no conflicts of interest.

## References

[1] Yuan Y., Zeng X.X., Zhao Y.F., Chen L.Q. (2017). Modified double-layer anastomosis for minimally invasive esophagectomy: an effective way to prevent leakage and stricture. World J Surg.

[2] Hayata K., Nakamori M., Nakamura M., Ojima T., Iwahashi M., Katsuda M. (2017). Circular stapling versus triangulating stapling for the cervical esophagogastric anastomosis after esophagectomy in patients with thoracic esophageal cancer: a prospective, randomized, controlled trial. Surgery.

[3] Awsakulsutthi S., Havanond C. (2015). A retrospective study of anastomotic leakage between patients with and without vascular enhancement of esophageal reconstructions with colon interposition: Thammasat University Hospital experience. Asian J Surg.

[4] Wang Z.Q., Jiang Y.Q., Xu W., Cai H.R., Zhang Z., Yin Z. (2018). A novel technique for cervical gastro-oesophageal anastomosis during minimally invasive oesophagectomy. Int J Surg.

[5] Goense L., van Rossum P.S., Tromp M., Joore H.C., van Dijk D., Kroese A.C. (2017). Intraoperative and postoperative risk factors for anastomotic leakage and pneumonia after esophagectomy for cancer. Dis Esophagus.

[6] Bolca C., Dumitrescu M., Fotache G., Stoica R., Cadar G., Cordos I. (2018). Comparative study of early postoperative complications: thoracic anastomosis vs cervical anastomosis – in esophageal replacement with gastric graft. Chirurgia (Bucur).

[7] van Rossum P.S., Haverkamp L., Carvello M., Ruurda J.P., van Hillegersberg R. (2017). Management and outcome of cervical versus intrathoracic manifestation of cervical anastomotic leakage after transthoracic esophagectomy for cancer. Dis Esophagus.

[8] Miro M., Farran L., Estremiana F., Miquel J., Escalante E., Aranda H. (2018). Does gastric conditioning decrease the incidence of cervical oesophagogastric anastomotic leakage?. Cir Esp.

[9] Yamana I., Takeno S., Yamada T., Sato K., Hashimoto T., Yamashita Y. (2017). The risk factors for refractory fistula after esophagectomy with gastric tube reconstruction in patients with esophageal cancer. Dig Surg.

[10] Mine S., Watanabe M., Okamura A., Imamura Y., Kajiyama Y., Sano T. (2017). Superior thoracic aperture size is significantly associated with cervical anastomotic leakage after esophagectomy. World J Surg.

[11] Li S.J., Wang Z.Q., Li Y.J., Fan J., Zhang W.B., Che G.W. (2017). Diabetes mellitus and risk of anastomotic leakage after esophagectomy: a systematic review and meta-analysis. Dis Esophagus.

[12] Goense L., van Rossum P.S., Ruurda J.P., van Vulpen M., Mook S., Meijer G.J. (2016). Radiation to the gastric fundus increases the risk of anastomotic leakage after esophagectomy. Ann Thorac Surg.

[13] Van Daele E., Van de Putte D., Ceelen W., Van Nieuwenhove Y., Pattyn P. (2016). Risk factors and consequences of anastomotic leakage after Ivor Lewis oesophagectomydagger. Interact Cardiovasc Thorac Surg.

[14] Biere S.S., Maas K.W., Cuesta M.A., van der Peet D.L. (2011). Cervical or thoracic anastomosis after esophagectomy for cancer: a systematic review and meta-analysis. Dig Surg.

[15] Tanaka K., Makino T., Yamasaki M., Nishigaki T., Miyazaki Y., Takahashi T. (2018). An analysis of the risk factors of anastomotic stricture after esophagectomy. Surg Today.

[16] Hanyu T., Kosugi S., Ishikawa T., Ichikawa H., Wakai T. (2015). Incidence and risk factors for anastomotic stricture after esophagectomy with gastric tube reconstruction. Hepatogastroenterology.

[17] Lindenmann J., Matzi V., Porubsky C., Anegg U., Sankin O., Gabor S. (2008). Self-expandable covered metal tracheal type stent for sealing cervical anastomotic leak after esophagectomy and gastric pull-up: pitfalls and possibilities. Ann Thorac Surg.

[18] Wu G., Yin M., Zhao Y.S., Fang Y., Zhao G., Zhao J. (2017). Novel esophageal stent for treatment of cervical anastomotic leakage after esophagectomy. Surg Endosc.

[19] Yin K., Xu H., Cooke D.T., Pu L.L. (2015). Successful management of oesophageal conduit necrosis by a single-stage reconstruction with the pedicled pectoralis major myocutaneous flap. Interact Cardiovasc Thorac Surg.

[20] Nakajima M., Satomura H., Takahashi M., Muroi H., Kuwano H., Kato H. (2014). Effectiveness of sternocleidomastoid flap repair for cervical anastomotic leakage after esophageal reconstruction. Dig Surg.

[21] Morita M., Ikeda K., Sugiyama M., Saeki H., Egashira A., Yoshinaga K. (2010). Repair using the pectoralis major muscle flap for anastomotic leakage after esophageal reconstruction via the subcutaneous route. Surgery.

[22] Hirao M., Yoshitatsu S., Tsujinaka T., Nishiyama A., Fujitani K., Nakamori S. (2006). Pectoralis myocutaneous flap with T-tube drainage for cervical anastomotic leakage after salvage operation. Esophagus.

[23] Williams J.K., Wood R.J., Hawes A., Mansour K.A. (1998). The use of the pectoralis myocutaneous flap for repair of a retrosternal esophagocolonic anastomotic leak. Plast Reconstr Surg.

[24] Heitmiller R.F., McQuone S.J., Eisele D.W. (1998). The utility of the pectoralis myocutaneous flap in the management of select cervical esophageal anastomotic complications. J Thorac Cardiovasc Surg.

[25] Liu M., Liu W., Yang X., Guo H., Peng H. (2017). Pectoralis major myocutaneous flap for head and neck defects in the era of free flaps: harvesting technique and indications. Sci Rep.

[26] Dent B., Griffin S.M., Jones R., Wahed S., Immanuel A., Hayes N. (2016). Management and outcomes of anastomotic leaks after oesophagectomy. Br J Surg.

[27] Wada T., Takeuchi H., Yoshikawa T., Oyama T., Nakamura R., Takahashi T. (2014). Successful management of anastomotic leakage and lung fistula after esophagectomy. Ann Thorac Surg.

[28] Schweigert M., Solymosi N., Dubecz A., Stadlhuber R.J., Muschweck H., Ofner D. (2013). Endoscopic stent insertion for anastomotic leakage following oesophagectomy. Ann R Coll Surg Engl.

[29] Kinoshita Y., Udagawa H., Tsutsumi K., Ueno M., Mine S., Ehara K. (2009). Surgical repair of refractory strictures of esophagogastric anastomoses caused by leakage following esophagectomy. Dis Esophagus.

[30] Gooszen J.A.H., Goense L., Gisbertz S.S., Ruurda J.P., van Hillegersberg R., van Berge Henegouwen M.I. (2018). Intrathoracic versus cervical anastomosis and predictors of anastomotic leakage after oesophagectomy for cancer. Br J Surg.

[31] Patel K., Lyu D.J., Kademani D. (2014). Pectoralis major myocutaneous flap. Oral Maxillofac Surg Clin North Am.

[32] Ariyan S. (1979). Further experiences with the pectoralis major myocutaneous flap for the immediate repair of defects from excisions of head and neck cancers. Plast Reconstr Surg.

[33] Teo K.G., Rozen W.M., Acosta R. (2013). The pectoralis major myocutaneous flap. J Reconstr Microsurg.

[34] Liu H.L., Chan J.Y., Wei W.I. (2010). The changing role of pectoralis major flap in head and neck reconstruction. Eur Arch Otorhinolaryngol.

[35] Guimaraes A.V., Aires F.T., Dedivitis R.A., Kulcsar M.A., Ramos D.M., Cernea C.R. (2016). Efficacy of pectoralis major muscle flap for pharyngocutaneous fistula prevention in salvage total laryngectomy: a systematic review. Head Neck.

