# Supplementary material for: Management of refractory cervical anastomotic fistula after esophagectomy using the pectoralis major myocutaneous flap
Source: Braz J Otorhinolaryngol. 2020 Jun 15;88(1):53–62. doi: 10.1016/j.bjorl.2020.05.009 (PMC9422472; doi:10.1016/j.bjorl.2020.05.009)

**Supplementary Figure 1** Severe adhesion and granulation tissue surrounding the fistula tract was found in exploration.


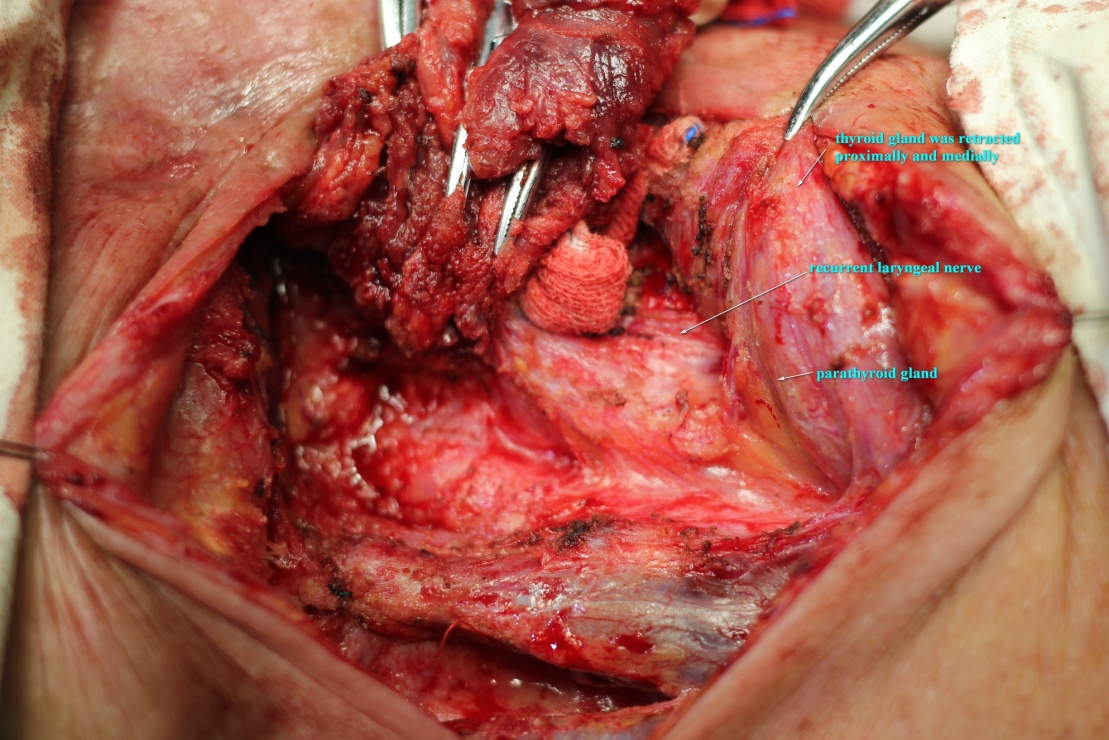


**Supplementary Figure 2** The anastomosis site was completely closed by stricture and accommodated only the nasogastric tube.


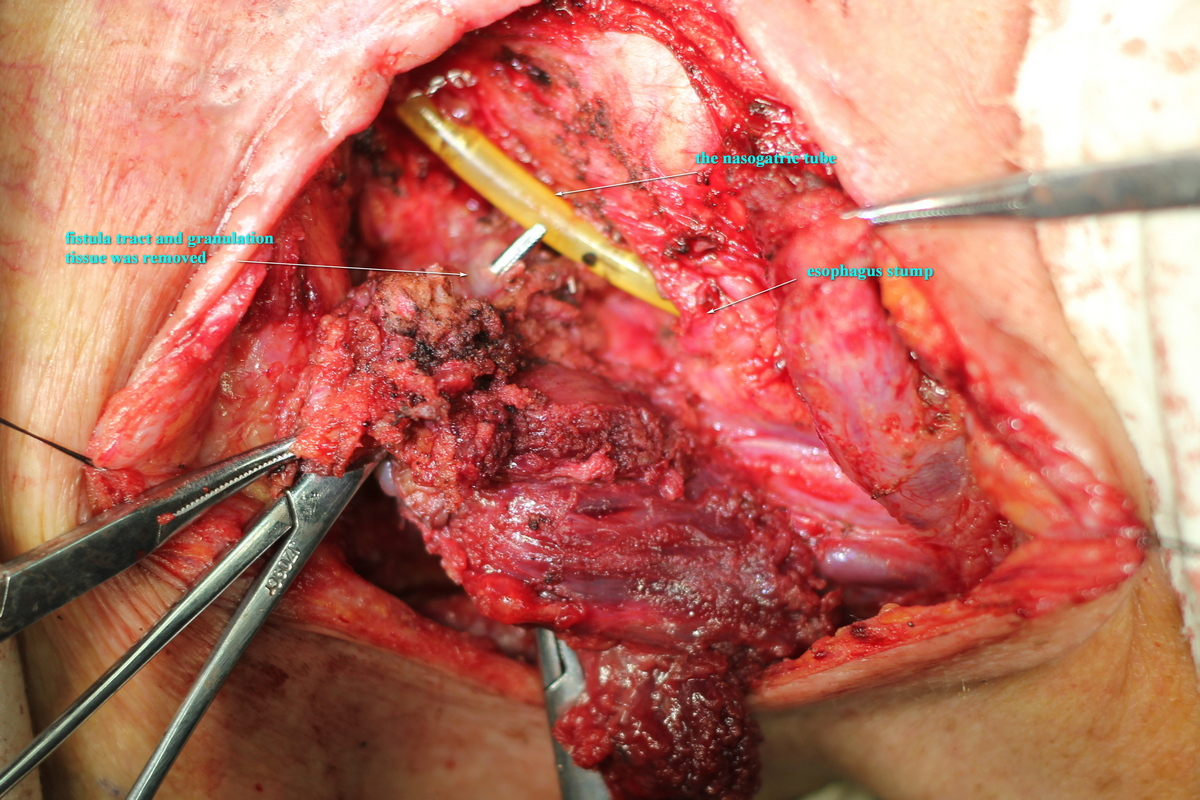


**Supplementary Figure 3** A PMF with skin paddle larger than previously designed (12×8 cm) was harvested and tubed to reconstruct the defect.


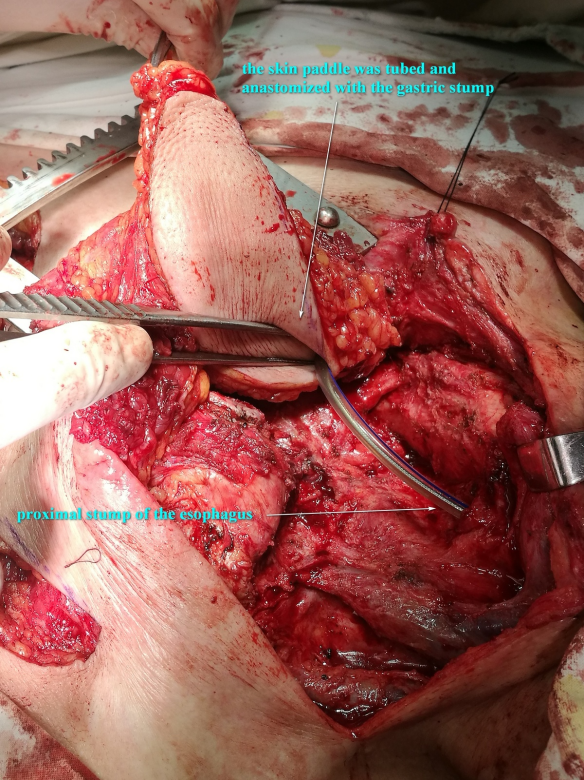


**Supplementary Figure 4** Six months after operation, barium contrast X-Ray demonstrated a mild anastomotic stricture with a diameter of 0.4 cm in the anastomotic site.


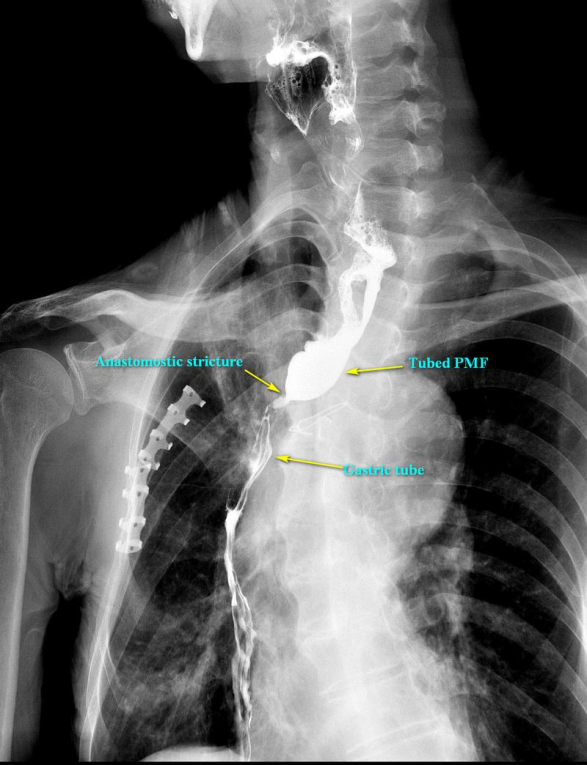

Supplement: Supplementary file 1 [file mmc1.docx]
